# Association between the *APOA2* rs3813627 Single Nucleotide Polymorphism and HDL and APOA1 Levels Through BMI

**DOI:** 10.3390/biomedicines8030044

**Published:** 2020-02-27

**Authors:** Hatim Boughanem, Borja Bandera-Merchán, Pablo Hernández-Alonso, Noelia Moreno-Morales, Francisco José Tinahones, José Lozano, Sonsoles Morcillo, Manuel Macias-Gonzalez

**Affiliations:** 1 Instituto de Investigación Biomédica de Málaga (IBIMA), Facultad de Ciencias, Universidad de Málaga, 29010 Málaga, Spain; h.b.boughanem@gmail.com; 2 Unidad de Gestión Clínica de Endocrinología y Nutrición del Hospital Virgen de la Victoria, Instituto de Investigación Biomédica de Málaga (IBIMA), Universidad de Málaga, 29010 Málaga, Spain; borjabandera@uma.es (B.B.-M.); pablo1280@gmail.com (P.H.-A.); fjtinahones@uma.es (F.J.T.); 3 Centro de Investigación Biomédica en Red de Fisiopatología de la Obesidad y la Nutrición, CIBERObn, 28029 Madrid, Spain; 4 Human Nutrition Unit, Faculty of Medicine and Health Sciences, Sant Joan Hospital, Institut d’Investigació Sanitària Pere Virgili, Rovira i Virgili University, 43201 Reus, Spain; 5 Department of Physiotherapy, School of Health Sciences, University of Malaga-Instituto de Investigación Biomédica de Málaga (IBIMA), 29010 Málaga, Spain; nmm@uma.es; 6 Departamento de Bioquímica y Biología Molecular, Facultad de Ciencias, Universidad de Málaga, 29010 Málaga, Spain; jlozano@uma.es

**Keywords:** rs3813627, APOA1, *APOA2*, HDL, BMI, SNP

## Abstract

*Background:* The interaction between obesity and genetic traits on high density lipoprotein (HDL) levels has been extensively studied. The variance of serum HDL has a strong genetic heritability, although the studied variant only explains a small part of this variation. The goal of this study was to investigate the associations between the apolipoprotein type 2 (*APOA2*) rs3813627 single nucleotide polymorphism (SNP) and anthropometric and biochemical variables, though body mass index (BMI). *Methods:* This study included 153 subjects (91 overweight/obese (BMI ≥ 25 kg/m^2^) and 62 non-obese individuals (BMI < 25 kg/m^2^)). The *APOA2* rs3813627 SNP was selected and genotyped. Genotype analysis was performed to analyze the associations between *APOA2* SNPs and anthropometric and biochemical variables through BMI. *Results:* The *APOA2* rs3813627 TT genotype was associated with low HDL levels in comparison with the *APOA2* rs3813627 GG and GT genotype in overweight/obese individuals, but not in the non-obese subjects (*p* < 0.05). The same trend was observed in the apolipoprotein type 1 (APOA1) protein levels (*p* < 0.05). Correlation analysis revealed a negative correlation between HDL and APOA1 levels and *APOA2* rs3813627 SNP under recessive model (*p* < 0.05). The odds ratio for low HDL levels was 3.76 and 3.94 for low APOA1 levels. The mediation analysis of *APOA2* rs3813627 SNP through BMI showed a full mediation on HDL and partial mediation on APOA1 levels (*p* < 0.05). Bioinformatic analysis showed that rs3813627 lies in the *APOA2* promoter and overlaps motifs for several bound transcription factors. *Conclusions*: On the basis of these data, the *APOA2* rs3813627 SNP is associated with low HDL and APOA1 levels susceptibility, and this effect was mediated by an increased BMI.

## 1. Introduction

Genome-wide association studies (GWAS) have been widely linked to several variants associated with various human traits such as circulating lipid levels, and have contributed to new biological insights into lipid traits and polymorphism interactions [1]. Multiple susceptibility loci have been associated with total cholesterol, triglycerides, low-density lipoprotein (LDL), and high-density lipoprotein (HDL) [2]. However, the majority of these genetic variants only explain a small part of the overall heritability for lipid traits. That is because a fraction of this missing heritability can be explained by gene-environment interactions, which are usually not included in the GWAS analysis [3]. Obesity constitutes one of the most important factors to alter lipid metabolism [4]. While the dual contribution of genetic and environmental factors to this relationship has been described in detail, its pathophysiological and genetic basis remains unclear [3]. HDL metabolism also depends on the genetic and environmental factors. According to family studies, serum HDL levels appear to be under a strong genetic basis. The heritability of circulating HDL has been estimated as being up to 80%, although the current genetic identified variants could only explain less than 10% of HDL variance, which leaves a wide gap of possibilities to new genetic variants [5]. Apolipoprotein A1 (APOA1) and apolipoprotein A2 (APOA2) are the major structural proteins of the HDL complex, which constitute about 70% and 20%, respectively. Both APOA1 and APOA2 play crucial physiological role in lipid storage, transport, and metabolism [6].

APOA2 is part of the apolipoprotein superfamily that includes the apolipoprotein A (APOA), apolipoprotein C (APOC), and apolipoprotein E (APOE) families. APOA2, encoded by the *APOA2* gene, is present as a homodimer in human plasma and as a monomer in murine plasma [1,2]. APOA2, the second most common apolipoprotein on the human HDL [7,8], has been considered of minor physiological importance in lipoprotein metabolism. A previous study reported that APOA2 deficiency has a minor influence on lipid and lipoprotein profiles or on the occurrence of coronary heart disease in human [9]. However, recent studies have shown that APOA2 plays multiple roles in the maintenance of HDL, obesity, and obesity-related insulin resistance [10,11]. Although the exact function of APOA2 is yet unclear, overexpression of the *APOA2* gene in mouse triggers an increase in body mass index (BMI) and triglycerides levels, whereas *APOA2*-null mouse decreases triglycerides levels by producing an altered HDL particles [12,13,14]. Moreover, the overexpression of murine *APOA2* gene in mice induces to larger HDL particles, whereas APOA2-deficient mice present decreased HDL particle size. Importantly, HDL particle size is associated with HDL levels, suggesting an association of APOA2 and circulating HDL levels [11,15,16].

In humans, the function of APOA2 also remains controversial. Indeed, all physiological and biochemical function data available have come from studies in animal models [4,14]. The *APOA2* gene has been associated with visceral fat accumulation and metabolism of triglyceride-rich lipoproteins [15]. It is also linked to obesity and lipid metabolism [11,16,17]. The APOA2 protein also participates in the regulation of lipoprotein metabolism enzyme by inhibiting the cholesterol ester transfer protein (CETP) (transport of cholesteryl esters and triglycerides between the lipoproteins), activating the lecithin-cholesterol acyltransferase (LCAT) (conversion of free cholesterol into cholesteryl ester), and phospholipid transfer protein (PLTP) (transfer phospholipids from triglyceride-rich lipoproteins to HDL), and modulating the hepatic lipase (LIPC) activity (hydrolysis of triacylglyceride) [10,13,18,19].

Nevertheless, only a few studies showed an association between *APOA2* polymorphisms and phenotypic effect in humans [17,18,19,20,21]. The most studied polymorphism is the *APOA2* rs5082 variant. This single nucleotide polymorphism (SNP) has been associated with waist circumference in men and abdominal fat depot in women [20,22]. In addition, rs5082 has been associated with BMI and food intake, and postprandial response to a saturated fat overload in healthy men [17,20]. On this basis, several SNPs have been reported, but only the *APOA2* rs3813627 SNP was associated with HDL concentration [19,23]. A previous study indicated that rs3813627 was considered as independent determinant of plasma HDL levels [24]. Another study showed that *APOA2* rs3813627 SNP was significantly different between participants with low and high HDL levels participants, and this SNP was identified as a susceptibility allele for low HDL-c and coronary heart disease risk [19]. However, both studies were conducted in only overweight/obese individuals and did not take into account the effect of the BMI on the HDL levels.

Considering the effect of the obesity and genetic basis of the *APOA2* rs3813627 SNP on HDL levels, we hypothesized that the genotype risk of this polymorphism could be associated with anthropometrical variables, glucose metabolism, and lipid traits. For that, we compared the non-obese with overweight/obese individuals according to their genotype on the anthropometrical variables, glucose, and lipid metabolism. Furthermore, we studied the mediator effect of BMI on HDL and APOA1 levels. This approach could serve us to evaluate whether this genetic variant is influencing on the lipid metabolism through BMI.

## 2. Materials and Methods

### 2.1. Participants and Study Design

This study included 153 participants and they were subdivided into two groups based on their BMI. Of these participants, 91 were overweight/obese (BMI ≥ 25 kg/m^2^) and 62 were non-obese (BMI < 25 kg/m^2^). The weight of all participants was stable in the last six months prior to participation in the study. The exclusion criteria were patients who had diabetes mellitus, acute or chronic inflammatory diseases, renal and infectious diseases, patients who had received treatment that altered their glucose metabolism such as metformin and lipid profile as statins, had altered other metabolic parameters, or consumed >20 g of ethanol/day. All participants were anonymized prior to typing and gave their written informed consent. The study was performed in accordance with the “Declaration of Helsinki” and approved by the Ethics Committees of “Virgen de la Victoria” University Hospital (Málaga, Spain).

### 2.2. Biochemical Determination

Blood samples were obtained from the antecubital vein and placed in ethylenediaminetetraacetic acid (EDTA) vacutainer tubes. Serum glucose, total cholesterol, triglycerides, and HDL were measured in a Dimension autoanalyzer (Dade Behring Inc., Newark, DE, USA) by enzymatic methods (Randox Laboratories Ltd., Ardmore, Crumlin, UK). Insulin was measured by radioimmunoassay (RIA) (BioSource International, Camarillo, CA, USA). LDL was calculated from the Friedewald equation. The measurement of HbA1c was performed by high performance liquid chromatography (Adams A1C, HA-8160, ARKRAY Kyoto, Japan), and the standardization was carried out according to the criteria of the National Glycohemoglobin Standardization Program [25]. The APOA1 circulating levels was measured by the immunoturbidimetric method according to the manufacturer’s instructions (DiaSys, Holzheim, Germany). Homeostasis model assessment of insulin resistance (HOMA-IR) was calculated with the following formula: HOMA-IR = fasting insulin (μIU/mL) × fasting glucose (mmol/L)/22.5 [26].

### 2.3. DNA Extraction and Genotyping

Genomic DNA was extracted from 200 μL peripheral blood by using a Qiamp DNA Blood mini kit (Qiagen GmbH, Hilden, Germany) according to the manufacturer’s instructions. DNA purity was measured by the A260/A280 and A260/A230 ratios. The information of the *APOA2* rs3613827 SNP was provided by the SNPedia Database System http://www.snpedia.com, dbSNP and Ensembl 2018 [27,28]. The SNP assays were performed using Allele Specific Quantitative PCR. The reaction mixture contained 10 µL 2X SYBR^®^ Premix Ex Taq™ II (TliRNaseH Plus) (Takara Bio, Inc., Madrid, Spain), 1 pM of each forward specific allele primers (wild type: 5′-GCGAATAGTTCTGCTAGA-3′ and mutant type: 5′-GCGAATAGTTCTGCTATA-3′) and common reverse primers (5′-TTCTGGAGGTCTACATCT-3′), 1 ng of template DNA, and nuclease free water making the final volume 20 µL. Polymerase chain reaction was performed using Eppendorf Mastercycler Realplex (Eppendorf, Madrid, Spain), under the following reaction conditions: 95 °C for 30 s followed by cycling for 40 cycles of denaturation at 95 °C for 5 s, and annealing and extension at 64 °C for 30 s. Cycle threshold (Ct) value and melt curve were used to determine the genotype of the sample. The allelic discrimination was verified using 1.5% agarose gel electrophoresis.

### 2.4. Bioinformatic Analysis

The predicted function of the rs3813627 *APOA2* SNP was examined by RegulomeDB, HaploReg, rSNPBase, SNP Function Prediction (FuncPred), PROMO software and PhenoScanner [29,30,31,32,33,34]. RegulomeDB is a database that includes relevant information about SNPs. This information shows predicted regulatory elements, DNase hypersensitivity, binding sites of transcription factors, and promoter regions. These SNP annotations are provided from gene expression omnibus (GEO), the encyclopedia of DNA element (ENCODE) project, and other published literatures. HaploReg is a tool for exploring variants associated with a phenotype, using linkage disequilibrium (LD) information, effect of regulatory motif and expression quantitative trait loci (eQTL) studies, and others. rSNPBase is a database of curated regulatory SNPs. FuncPred was used to study the regulatory potential of SNPs. This tool uses the TRANSFAC database on potential transcription factor recognition sites. We have also examined the association between the rs3813627 *APOA2* SNP and the expression levels of *APOA2* in many human tissues in the genotype tissue expression (GTEx) portal. PhenoScanner is a curated database holding publicly available results from large-scale genome-wide association studies. This database provides relationships between genetic variants with a broad range of phenotypes, as gene expression.

### 2.5. Statistical Analyses

Continuous variables are shown as the mean and standard deviation. Normality was assessed with the Kolmogorov–Smirnov test. Differences between qualitative variables and the Hardy–Weinberg equilibrium (HWE) were tested using χ^2^ test. The comparisons between non-obese and overweight/obese individuals were made using Welch’s two sample *t*-test for variables following a normal distribution. The comparisons between genotypes for parametric variable were performed using Welch’s two sample *t*-test as we found unequal variances and differing sample size. The Kruskal–Wallis test was used to evaluate the difference between groups for variables not following a normal distribution. Pearson’s correlation coefficients were calculated to evaluate the association between the study variables in the whole population. *Odds ratio* (OR) and 95% confidence intervals (CI) were estimated by multivariant logistic regression models to assess the association of the *APOA2* rs3813627 TT risk genotype with low HDL and APOA1 levels in the overweight/obese group. Model 1 was carried out without adjustment and Model 2 was adjusted by sex. The *APOA2* rs3813627 GG genotype was considered as the reference category. Lastly, we assessed whether the *APOA2* rs3613827 TT risk genotype was associated with low levels of HDL and APOA1, and whether this association was mediated by BMI. To test this, we conducted a causal mediation analysis, testing whether this association significance between *APOA2* rs3613827 SNP and HDL and APOA1 levels was mediated via BMI, on the overweight/obese individuals. Significance of the mediation effect was performed using 5000 bootstrapped iterations mean indirect and direct effect, where the model was adjusted by sex. Statistical analyses were performed using RStudio software version 2.8.1. [35].

## 3. Results

### 3.1. Characteristics of the Study Population and Genotype Frequencies

The baselines characteristics of the non-obese (*n* = 62) and overweight/obese groups (*n* = 91) are shown in Table 1. While there were no significant differences in age, sex, total cholesterol, and LDL between both groups, overweight/obese subjects showed significantly increased values of anthropometric variables as weight, waist, hip, waist-to-hip ratio (WHR), and BMI as compared with the non-obese individuals (*p* < 0.05). The overweight/obese group also showed significantly higher values of fasting glucose, glycated hemoglobin (Hb1Ac), fasting insulin, HOMA-IR, and triglycerides, and s significantly decreased value of HDL as compared with the non-obese individuals (*p* < 0.05).

The frequencies of the *APOA2* rs3813627 SNP of all subjects, non-obese and overweight/obese individuals, are summarized in Table 2. The rs3813627 allele and genotype distributions did not deviate from the Hardy–Weinberg equilibrium for all groups (*p* = 0.22 for all subjects, *p* = 0.42 for non-obese individuals and *p* = 0.35 for overweight/obese individuals). The minor allele frequencies of the *APOA2* rs3813627 polymorphism were 0.36 for the whole population, 0.37 for the non-obese group, and 0.35 for overweight/obese group. Non-significant differences were found among the genotype and allele frequencies according to the non-obese and overweight/obese groups (Table 2).

### 3.2. Analysis of the Association between the APOA2 rs3813627 SNP and HDL and APOA1 Levels

In a first step, we tested whether the *APOA2* rs3813627 SNP was associated with anthropometric variables, glucose, and lipid metabolism and compared the *APOA2* rs381362 risk genotype with the normal *APOA2* rs3813627 genotypes. The complete analysis comparing the GG, GT, GG + GT versus TT allele of all subjects, non-obese, and overweight/obese individuals is summarized in the Table 3 and Table 4.

**Figure 1:**
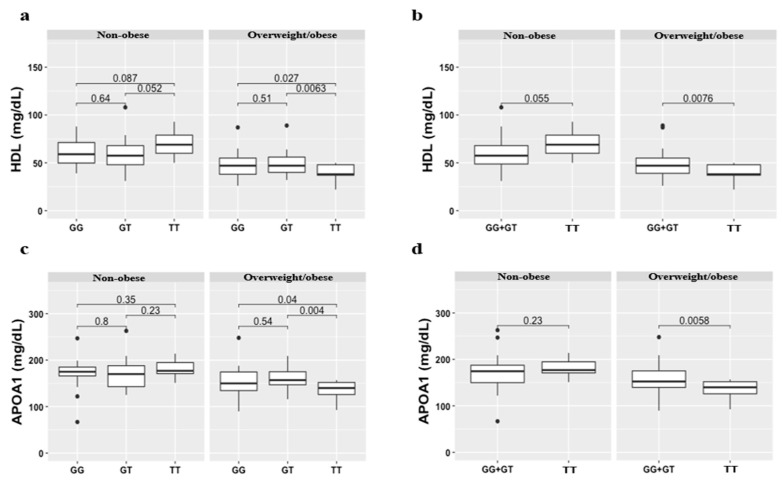
High density lipoprotein (HDL) and APOA1 levels according to the *APOA2* rs3813627 genotypes in non-obese and overweight/obese individuals. (**a**,**b**) Representation of HDL levels according to the *APOA2* rs3813627 genotype in non-obese (*n* = 62) (BMI < 25 kg/m^2^) (GG = 26, GT = 26, GG + GT = 54, and TT = 10) and overweight/obese (*n* = 91) (BMI ≥ 25 kg/m^2^) individuals (GG = 41, GT = 37, GG + GT = 78, and TT = 13); (**c**,**d**) Representation of APOA1 levels according to the *APOA2* rs3813627 genotype in non-obese (*n* = 52) (GG = 21, GT = 23, GG + GT = 44, and TT = 8) and overweight/obese individuals (*n* = 65) (GG = 27, GT = 25, GG + GT = 52, and TT = 13). Results are presented as means ± S.D. *p*-value indicates significant difference between genotypes according to Welch’s two sample *t*-test (*p*  <  0.05). Abbreviations: HDL, high density lipoprotein; APOA1, apolipoprotein type A1; APOA2, apolipoprotein type A2.

Overall, we observed that the *APOA2* rs381362 risk genotype (genotype TT) was associated with low levels of HDL in the overweight/obese individuals. The HDL value of individuals that had the TT genotype was significantly decreased (40.08 mg/dL) as compared with individuals carrying the GG (46.85 mg/dL), GT (48.59 mg/dL), and GG + GT (47.68 mg/dL) genotype (*p* < 0.05) (Figure 1a,b). However, no significant differences were observed for the HDL value in the non-obese individuals that had the TT genotype as comparing with individuals carrying the GG, GT, and GG + GT genotype (Figure 1a,b and Table 4). Moreover, we also observed that the *APOA2* rs381362 risk genotype was associated with low levels of APOA1 in the overweight/obese individuals. The APOA1 levels of individuals that had the TT genotype was significantly decreased (136.58 mg/dL) as compared with individuals carrying the GG (153.19 mg/dL), GT (157.84 mg/dL), and GG + GT (155.43 mg/dL) genotype (*p* < 0.05) (Figure 1c,d and Table 4). When compared the risk and normal genotypes in non-obese individuals, we did not find significant differences in APOA1 levels (Figure 1c,d, Table 3, and Table 4).

**Figure 2:**
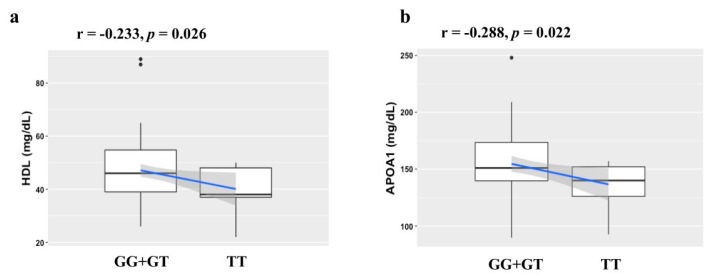
Correlation analysis between the *APOA2* rs3813627 SNP and HDL and APOA1 levels. Correlation between the *APOA2* rs3813627 polymorphism and (**a**) HDL (*n* = 91) and (**b**) APOA1 (*n* = 65) levels under recessive model (GG + GT and TT) in overweight/obese (BMI ≥ 25 kg/m^2^) individuals according to the Pearson’s correlation test (*p*  <  0.05). Abbreviations: BMI, body mass index; HDL, high density lipoprotein; APOA1, apolipoprotein type A1; APOA2, apolipoprotein type A2.

This relationship between HDL and APOA1 levels and the *APOA2* rs3813627 SNP was also evaluated through Pearson′s correlation analysis in the overweight/obese individuals using rs3813627 recessive model (GG + GT and TT). Under recessive model, the *APOA2* rs3813627 variant was significantly correlated with HDL (r = −0.233 and *p* = 0.026) and APOA1 (r = −0.284 and *p* = 0.022) levels under a negative correlation (Figure 2a,b).

We further analyzed the association between the *APOA2* rs3813627 risk genotype and the risk of low value of HDL and APOA1 under four genetic models (overdominant, dominant, recessive, and additive) in overweight/obese individuals using multivariant logistic regression analysis to estimate the OR and 95% CI of this association. *APOA2* rs3813627 SNP was not associated with BMI in any model and after adjusting by sex (data not shown). However, we observed that this polymorphism was associated with low HDL levels under recessive model (genotype TT: OR = 3.76, CI 95% 1.11–12.71, *p* = 0.030). After adjusting by sex, this association remained statistically significant (genotype TT: OR = 3.76, CI 95% 1.09–13.02, *p* = 0.033). We also found that rs3813627 was significantly associated with an increased risk of low APOA1 levels, under recessive model (genotype TT: OR = 3.89, CI 95% 1.10–13.81, *p* = 0.035) and after adjusting by sex (genotype TT: OR = 3.94, CI 95% 1.11–14.80, *p* = 0.034). In the codominant, dominant and log-additive model, we observed that these models were not significantly associated with low HDL and APOA1 levels (Table 5).

### 3.3. The Mediation Effect of the BMI on HDL and APOA1 Levels

**Figure 3:**
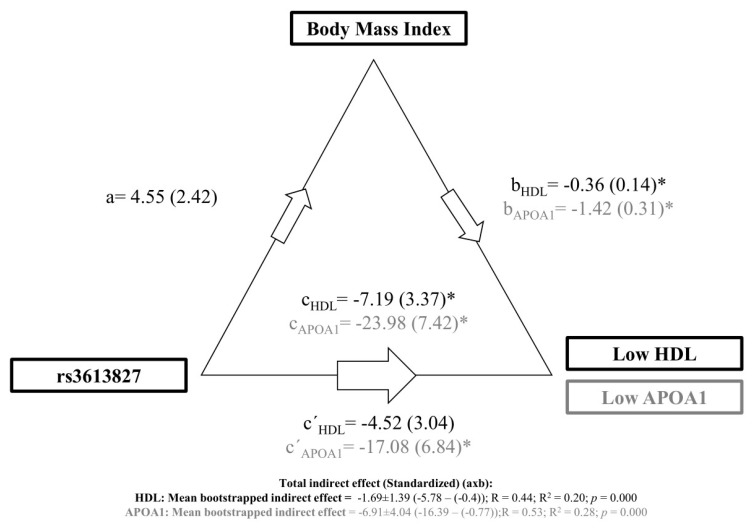
Causal mediation model of the total effect of *APOA2* rs3813627 on HDL and APOA1 levels through BMI. The mediation effect of *APOA2* rs3813627 risk genotype carriage on low HDL and APOA1 levels through BMI (assessed on the overweight/obese individuals, *n* = 95 subjects for HDL and *n* = 65 for APOA1) after adjusting by sex. Path-weights are displayed as beta values with standard errors in brackets. Asterisks indicate significant *p*-values (* *p* < 0.05). Significance of the indirect effect was determined using mean bootstrapped. (a) Direct effect of rs3813627 on BMI; (b) Direct effect of BMI on parameters (HDL and APOA1 levels); (c) Total effect of rs3813627 on parameters (HDL and APOA1 levels) through BMI; (c**´**) Direct effect of rs3813627 on parameters (HDL, and APOA1 levels) removing BMI. Abbreviations: BMI, body mass index; HDL, high density lipoprotein; APOA1, apolipoprotein type A1.

To clarify the relationship between the *APOA2* rs3813627 SNP, low HDL, and APOA1 levels and BMI, we tested whether TT risk genotype in the overweight/obese individuals was associated with low HDL and APOA1 levels and whether these effects were mediated via BMI. To test this idea, we applied causal mediation analysis. The significance of the mediation effect was determined using bootstrapping iterations, where each path of the model was adjusted by sex (Figure 3). We found in this model that the *APOA2* rs3813627 SNP was not significantly associated with BMI (a, as direct effect of rs3813627 on BMI: a = 4.55 ± 2.42, *p* = 0.063). In addition, BMI was significantly associated with low HDL levels (b, as direct effect of BMI on HDL levels: b_HDL_ = −0.36 ± 0.14, *p* = 0.013). We observed that the total effect (c, as total effect of rs3813627 on HDL through BMI) was significantly associated (c_HDL_ = −7.19 ± 3.22, *p* = 0.027). However, the *APOA2* rs3813627 SNP was not associated with low HDL levels removing the effect of the BMI (c´, as direct effect of rs3813627 on HDL removing BMI: c´_HDL_ = −4.53 ± 3.04, *p* = 0.090). Supporting our results, the analyses revealed a significant mediating effect with BMI as mediator, and *APOA2* rs3813627 SNP as predictor on the HDL levels (20% of the mediation effect, index = −1.69 ± 1.39 (CI: −5.78–(−0.4)), *p* = 0.000). The effect was considered a full mediation, since the direct effect of the *APOA2* rs3813627 risk genotype on HDL was not significant (see c´) in the absence of the mediator.

On the other hand, BMI was negatively associated with low APOA1 levels (b, as direct effect of BMI on APOA1 levels: b_APOA1_ = −1.42 ± 0.31, *p* = 0.000). The total effect of the *APOA2* rs3813627 SNP through BMI on low APOA1 levels was significantly associated (c, as total effect of rs3813627 on APOA1 through BMI: c_APOA1_ = −23.98 ± 7.42, *p* = 0.002). Moreover, rs3813627 was significantly associated with low APOA1 levels without the effect of the BMI (c´, as direct effect of rs3813627 on APOA1 removing BMI: c´_APOA1_ = −17.08 ± 6.84, *p* = 0.014). Summarizing these results, the analyses revealed a significant mediating effect with BMI as mediator, and *APOA2* rs3813627 SNP as predictor on the APOA1 levels (28% of the mediation effect, index = −6.91 ± 4.04 (CI: −16.39–(−0.77)), *p* = 0.000). The effect was considered a partial mediation, since the direct effect of the *APOA2* rs3813627 risk genotype on HDL was significant (see c´) in the absence of the mediator.

### 3.4. Bioinformatic Analysis: rs3813627 Is Likely to Affect Binding to Transcription Factors

**Figure 4:**
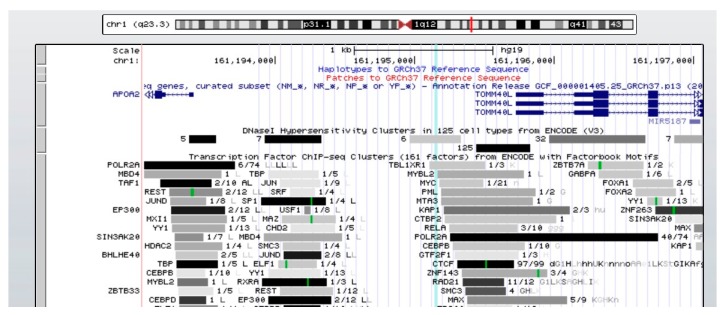
The position of rs3813627 is highlighted in light blue, showing that rs3813627 is in the promoter region of APOA2 and overlaps several DNase hypersensitivity clusters and TBL1XR1 transcription factor determined by experimental procedures (ENCODE project). The red bar shows the position of the SNP on the chromosome. The blue color shows the position of the genes. The black and gray color show the binding sites of the transcription factors throughout the sequence.

The rs3813627 *APOA2* SNP is a noncoding variant, since it is situated in the promoter region of the *APOA2* gene (Figure 4). We examined the potential functional effect of this SNP in various databases. RegulomeDB uses a score system ranging from 1 to 7, where a score of 1 shows the strongest evidence of affect to binding to transcription factors. A score of 7 shows the least evidence of being SNP functional. RegulomeDB assigns rs3813627 a score of 2a. The score 2a represents that rs3813627 is related to transcription factor binding, any motif, DNase footprints, and DNase sensitivity, which means that there is binding evidence to likely affect binding to transcription factors, and it is related to functional consequences. In addition, rs3813627 lies in the promoter of *APOA2* and overlaps motifs for several bound transcription factors including transducin beta-like 1 X-linked receptor 1 (TBL1XR1), androgen receptor (AR), and heat shock transcription factor 1 (HSF1) (Table 6 and Figure 4). Using HaploReg, we found that there are six SNPs in LD (r^2^ > 0.8) with rs3813627. These SNPs also overlap promoter marks, DNase sensitivity, and enhancer marks for several cell lines. Between them, there is a SNP that overlaps a transcription factor binding sites, as rs3829793, a SNP also situated in the *APOA2* promoter, and rs3829793 is a candidate as it overlaps regulatory marks in the most cell lines. It also disrupts several motifs and binds various transcription factors.

In addition to RegulomeDB and HaploReg, we also examined rs3813627 in the rSNPBase and GTEx portal; rSNPBase confirmed that rs3813627 was in LD with several SNPs and was related with proximal regulation of *APOA2*, through binding with several transcription factors. In addition, rSNPBase and GTEx portal reported that rs3813627 was candidate to be an eQTL for *APOA2* (Table 5). Furthermore, rs3813627 was also related with *APOA2* expression, as suggests the PhenoScanner tool. Data about all of the database is summarized in Table 6.

## 4. Discussion

Multiple epidemiological studies have shown that low levels of HDL and APOA1 are considered an independent and traditional risk factor of several diseases including coronary artery diseases, metabolic syndromes, dyslipidemias, and multiple cancer types [43,44,45,46,47]. During the last few years, great efforts have been made to discover new candidate genes and several genetic variations that determine the HDL levels. However, the role of several of these genes and variants remains unknown [5]. Moreover, HDL is a complex trait that involves interactions between multiple genes that interact with each other and with their environmental factors.

The main finding of this study was that this is the first evidence that the *APOA2* rs3813627 polymorphism was associated with low HDL and APOA1 levels, in overweight/obese individuals, but this association was not found in the non-obese individuals. Our results are consistent with previous studies conducted in the Veterans Affairs HDL Intervention Trial (VA-HIT) and controls selected from the Framingham Offspring Study (FOS) in the American population. These studies showed an association between susceptibility TT genotype of the *APOA2* rs3813627 variant and low HDL-c levels related to coronary heart diseases, but did not include the BMI as a risk factor, as we have shown in our results [19,24]. Within the overweight/obese participants, a significant association was found between reduced HDL and APOA1 levels and the TT genotype. In this study, the *APOA2* rs3813627 risk genotype had an increased risk effect of low HDL (OR = 3.76 and *p* = 0.033) and APOA1 levels (OR = 4.94 and *p* = 0.034) levels. Our findings support the hypothesis that *APOA2* rs3813627 SNP is associated with low HDL levels. However, we have contributed by suggesting that the BMI could be involved in an effect/interaction/association between the genetic basis of the *APOA2* rs3813627 SNP to determine the susceptibility of the HDL metabolism. These data are also interesting from a genetic variant perspective, highlighting a strong association with the capacity of the genetic basis of the HDL levels, and also providing new insights into the gene-environmental analysis.

Indeed, APOA2 plays a complex role in lipoprotein metabolism and HDL size and consecutively concentrations. The exact function is still uncertain but it can increase the structural stability of the HDL particles and participates in the determination of the correct and functional size of the HDL complex. This stability is achieved, in part, by its participation in the modulation of the metabolic enzymes activity and its ability to compete for the HDL surface with APOA1 [7,13,48,49]. Additionally, previous studies have demonstrated that the variation of the *APOA2* gene expression was associated with the size and heterogeneity of the HDL particles, suggesting that HDL particle size depends on APOA2 concentrations [15,16].

The expression of *APOA2* gene and the correlated APOA2 protein quantity in plasma could explain the HDL levels because of the heterogeneity of the size and quantity of the HDL particles. Our second major finding was that the association between the *APOA2* rs3813627 variant and low HDL and APOA1 levels was mediated via BMI. This suggests that the *APOA2* rs3813627 risk genotype contributes to reduced HDL and APOA1 levels in overweight/obese individuals, resulting in an increased risk and susceptibility to disorders related to low HDL and APOA1 levels. Our mediation analysis also suggests that there is a novel mechanism underlying the established association between HDL and BMI in large epidemiological studies. However, many determinants were not clarified because genetic variants only explain a small fraction of HDL variance. Here, we found a novel strategy in which an increased BMI has a full mediation effect of reduced HDL and a partial mediation effect on the APOA1 levels and the *APOA2* rs3613827 variant as predictor. Still, it is unclear whether APOA2 is a cause or consequence of low HDL and APOA1 levels through BMI. Our current results suggest that the *APOA2* rs3813627 risk genotype is associated with low HDL and APOA1 levels, but an increased BMI seems to have an interesting effect of low HDL and APOA1 levels when the risk genotype is present.

The mechanism by which rs3613827 affects HDL-c metabolism and levels is still unknown. The transcription of the *APOA2* gene is controlled by regulatory elements in the promoter region, located between nucleotides −903 to −33 with respect to the transcription start site. The promoter regulatory elements can be categorized as four proximal (A to CD), four middle (D to H), and six distal (I to N) elements, but the liver-specific expression of *APOA2* gene is mediated by the heat-stable protein upstream stimulatory factors (USF) 1 and 2, the CCAAT enhancer-binding protein (C/EBP), and the heat-labile factor AIIABI transcription factors [50,51,52]. The bioinformatic analysis performed in this study revealed that the variant site of the rs3813627 polymorphism, situated upstream at the position −1730 from the *APOA2* gene promoter, is a binding site for multiple transcription factors. The fact that rs3813627 mediates the binding of several transcription factors could indicate its role as a regulatory element, in turn, resulting in changes in gene expression of APOA2. Nevertheless, experimental assays are needed to confirm this bioinformatic analysis. The molecular mechanism underlying the association between *APOA2* SNP variants and low HDL and APOA1 levels are not yet known. With respect to the link between APOA2 and HDL levels, previous studies have been carried out in *APOA2* rs5082 SNP and showed a decreased expression profile of up to 30% in gene expression and lower plasma concentrations of APOA2 as compared with wild genotypes. In addition, rs5082 was involved in the regulation of dietary intake and a lower postprandial response. These findings can partially explain the role of APOA2 in the metabolism of lipids in obese patients and can identify a population at risk to mitigate the gene effect of risk polymorphisms [17,23] (Table 7).

Although the current findings suggest that *APOA2* rs3813627 was associated with low HDL and APOA1 levels, we caution that other mechanisms could contribute to an increased risk of decreased levels of HDL and APOA1. The variability of the concentrations and heterogeneity of HDL in overweight/obese individuals could be the result of a number of metabolic changes [53]. These changes could be produced by increasing fractional clearance of HDL secondary to reduced cholesterol content, and reducing production of the main apoprotein in HDL complex, such as APOA1 and APOA2 [54]. The effect of obesity on HDL and APOA1 levels could be modulated by lipid and glucose metabolism from dietary consumption. The quantity of these nutrients could modulate the gene expression of APOA1, and therefore, specific nutrients could have a significant effect on plasma HDL concentration. The role of APOA2 in obesity could be explained by promoting and inhibiting cholesterol efflux controlled by the enzymatic activity. In fact, the concentration of plasma APOA2 in overweight/obese individual could partially explain the variability of size and concentration of HDL particles. Understanding the mechanisms of low HDL in obesity would help in the development of interventions that could reduce the risk of low-HDL and associated metabolic disease in individuals with an increased BMI.

Regarding limitations of our research, it is necessary to replicate the study in other cohorts and validate the results through experimentation, measuring the size of the HDL particles, the APOA2 plasma concentration, and the rate of *APOA2* gene expression, as well as increase the sample size of our population study. Despite all these limitations, this study was a singular and first attempt to investigate the relation between HDL and *APOA2* polymorphism in overweight/obese individuals. Our data could be helpful for detecting high-risk genotypes, designing screening programs, and providing dietary suggestions to prevent low HDL in overweight and obese. Although the retrospective nature of this study generates hypothesis, it is also one of its limitations and further mechanistic studies are required in order to more clearly determine an association versus true causality of the *APOA2* variant (genotype TT) on HDL-c concentrations. Finally, given the characteristics of our cohort, the data presented in this study did not adjust for other factors that alter the plasma concentrations of HDL, such as the type of diet, physical exercise, or smoking.

## 5. Conclusions

In conclusion, we have shown that the presence of the TT genotype of the *APOA2* rs3813627 polymorphism was associated with an increased risk of low HDL and APOA1 levels in overweight/obese individuals. This effect was fully mediated by BMI for HDL and partially mediated by BMI for APOA1 levels. The effect of rs3813627 could affect the variation of *APOA2* expression rate, however, future studies are needed to confirm these observations.

## Figures and Tables

**Table 1:** General data and characteristics of all subjects.

Variables	All Subjects	Non-Obese (BMI < 25 kg/m^2^)	Overweight/Obese (BMI ≥ 25 kg/m^2^)
*n*	153	62	91
Sex (male/female)	70/83	26/36	44/47
(%)	46/54	42/58	48/52
Weight (kg)	82.89 ± 24.12	63.96 ± 9.08	96.77 ± 22.19 *
Waist (cm)	99.83 ± 24.12	82.77 ± 8.43	110.63 ± 16.26 *
Hip (cm)	104.76 ± 22.81	93.21 ± 8.28	112.07 ± 25.90 *
WHR	1.01 ± 0.46	0.89 ± 0.07	1.09 ± 0.57 *
BMI (kg/m^2^)	29.93 ± 8.62	22.95 ± 1.69	34.69 ± 8.18 *
Fasting glucose (mg/dL)	99.14 ± 14.87	93.16 ± 9.89	103.15 ± 16.28 *
Hb1Ac (%)	5.65 ± 0.48	5.41 ± 0.38	5.64 ± 0.51 *
Fasting insulin (µIU/mL)	11.32 ± 10.09	6.67 ± 4.63	14.37 ± 11.47 *
HOMA-IR	2.94 ± 2.99	1.56 ± 1.20	3.85 ± 3.43 *
Total cholesterol (mg/dL)	199.59 ± 34.98	198.16 ± 33.15	200.55 ± 36.30
Triglycerides (mg/dL)	122.47 ± 64.75	94.03 ± 45.45	141.53 ± 68.86 *
HDL (mg/dL)	52.18 ± 14.60	60.51 ± 14.89	46.59 ± 11.46 *
LDL (mg/dL)	125.52 ± 32.10	119.82 ± 28.57	129.34 ± 33.89

Results are presented as means ± S.D. Asterisks indicate significant differences between non-obese (BMI < 25 kg/m^2^) and overweight/obese (BMI ≥ 25 kg/m^2^) individuals according to Welch’s two sample *t*-test for parametric variables and the Chi-square test for sex variables expressed as percentage (* *p* < 0.05). Abbreviations: WHR, waist-to-hip ratio; BMI, body mass index; HbA1c, glycated hemoglobin; HOMA-IR, homeostasis model assessment of insulin resistance; HDL, high density lipoprotein; LDL, low density lipoprotein.

**Table 2:** Genotypic and allelic frequencies *APOA2* rs3813627 polymorphism.

rs3813627	All Subjects (%)	Controls (BMI < 25 kg/m^2^) (%)	Overweight/Obese (BMI ≥ 25 kg/m^2^) (%)	*p* ^a^
GG genotype	43.79	41.93	45.05	0.91
GT genotype	41.18	41.93	40.66	
TT genotype	15.03	16.13	14.28	
G allele	64.37	62.90	65.38	0.66
T allele	35.62	37.10	34.61	
*p* ^b^	0.22	0.42	0.35	

Results are given as percentages of the genotype and allele frequencies. The Chi-square test was performed to determine significant difference between genotype groups, *p*^a^ shows the genotype differences between non-obese and overweight/obese individuals, and *p*^b^ shows whether the genotype frequencies of the *APOA2* rs3813627 variant in different groups are in the Hardy–Weinberg equilibrium. (*p* < 0.05). Abbreviations: BMI, body mass index.

**Table 3:** General data of all subjects according to the *APOA2* rs3813627 genotypes.

Variables	All Subjects			
Genotypes	GG	GT	GG + GT	TT
*n*	67	63	130	23
Weight (kg)	82.64 ± 22.34	81.93 ± 24.28	82.21 ± 23.20	86.06 ± 28.39
Waist (cm)	99.39 ± 16.92	99.94 ± 20.70	99.65 ± 18.71	100.78 ± 22.88
Hip (cm)	107.04 ± 15.32	107.31 ± 19.46	107.75 ± 17.31	108.65 ± 20.85
WHR	1.07 ± 0.58	0.98 ± 0.37	1.03 ± 0.49	0.92 ± 0.06 ^**a,c**^
BMI (kg/m^2^)	29.63 ± 7.44	29.69 ± 9.13	29.66 ± 8.27	31.43 ± 10.43
Fasting glucose (mg/dL)	100.26 ± 16.62	99.94 ± 12.50	99.45 ± 14.73	97.32 ± 15.88
HB1Ac (%)	5.46 ± 0.48	5.64 ± 0.41	5.54 ± 0.45	5.53 ± 0.56
Fasting insulin (µIU/mL)	11.21 ± 10.22	10.49 ± 9.45	10.86 ± 9.82	14.10 ± 11.48
HOMA-IR	2.96 ± 3.06	2.67 ± 2.64	2.82 ± 2.85	3.68 ± 3.65
Total cholesterol (mg/dL)	197.57 ± 40.47	202.76 ± 30.64	200.08 ± 36.00	196.68 ± 28.70
Triglycerides (mg/dL)	121.36 ± 64.89	127.75 ± 59.22	121.31 ± 61.97	129.27 ± 80.62
HDL (mg/dL)	51.91 ± 13.98	52.46 ± 14.10	52.17 ± 13.99	52.18 ± 18.20
LDL (Friedwald)	124.12 ± 37.30	127.75 ± 27.58	125.88 ± 32.87	123.41 ± 27.65

Results are presented as means ± S.D. Significant results are presented as bold. The *p*-value indicates significant difference between (a) GG vs. TT individuals, (b) GT vs. TT individuals, and (c) GG + GT vs. TT individuals according to the Welch’s two sample *t*-test (*p*  <  0.05). Abbreviations: WHR, waist-to-hip ratio; BMI, body mass index; HbA1c, glycated hemoglobin; HOMA-IR, homeostasis model of insulin resistance; HDL, high density lipoprotein; LDL, low density lipoprotein.

**Table 4:** General data of all subjects according to the *APOA2* rs3813627 genotypes and body mass index (BMI).

Variables	Controls BMI < 25 kg/m^2^					Overweight/Obese BMI ≥ 25 kg/m^2^		
Genotypes	GG	GT	GG + GT	TT	GG	GT	GG + GT	TT
*n*	26	26	52	10	41	37	78	13
Weight (kg)	65.8 ± 9.12	62.95 ± 10.09	64.51 ± 9.56	61.48 ± 6.09	95.30 ± 21.08	96.79 ± 22.62	95.05 ± 21.68	104.96 ± 23.64
Waist (cm)	84.44 ± 8.78	82.41 ± 8.81	83.49 ± 8.76	79.40 ± 5.89	108.51 ± 13.94	110.66 ± 18.43	109.52 ± 16.12	117.23 ± 16.11
Hip (cm)	96.00 ± 7.37	91.68 ± 8.43	93.98 ± 8.09	89.60 ± 8.63	113.78 ± 15.04	116.86 ± 18.08	115.22 ± 16.48	123.31 ± 14.36
WHR	0.88 ± 0.07	0.90 ± 0.06	0.89 ± 0.07	0.89 ± 0.05	1.19 ± 0.71	1.02 ± 0.47	1.11 ± 0.06	0.95 ± 0.06 ^**a,c**^
BMI (kg/m^2^)	23.45 ± 1.25	22.75 ± 2.03	23.10 ± 1.71	22.13 ± 1.43 ^a^	33.55 ± 7.06	34.57 ± 9.03	34.04 ± 8.01	38.58 ± 8.39
Fasting glucose (mg/dL)	93.57 ± 9.93	94.08 ± 10.73	93.82 ± 10.24	89.33 ± 6.78	104.51 ± 18.61	101.75 ± 12.81	103.20 ± 16.08	102.85 ± 18.16
Hb1Ac (%)	5.35 ± 0.38	5.53 ± 0.40	5.43 ± 0.39	5.28 ± 0.32	5.54 ± 0.54	5.72 ± 0.40	5.63 ± 0.48	5.71 ± 0.63
Fasting insulin (µIU/mL)	7.56 ± 6.31	6.13 ± 2.92	6.83 ± 4.89	5.63 ± 2.31	13.50 ± 11.53	13.57 ± 11.14	13.53 ± 11.27	19.31 ± 11.81
HOMA-IR	1.81 ± 1.67	1.42 ± 0.69	1.61 ± 1.27	1.23 ± 0.51	3.68 ± 3.50	3.54 ± 3.13	3.62 ± 3.31	5.19 ± 3.95
Total cholesterol (mg/dL)	200.38 ± 37.20	196.27 ± 33.05	198.32 ± 34.90	197.22 ± 21.89	197.78 ± 42.77	207.32 ± 28.41	201.25 ± 36.89	196.31 ± 33.49
Triglycerides (mg/dL)	96.77 ± 44.51	102.06 ± 50.93	97.57 ± 47.37	73.55 ± 25.27 ^a^	136.95 ± 71.18	137.35 ± 59.95	137.96 ± 65.67	167.85 ± 83.71
HDL (mg/dL)	59.88 ± 13.33	57.96 ± 15.99	58.92 ± 14.61	69.67 ± 13.83	46.85 ± 11.99	48.49 ± 11.28	47.68 ± 11.62	40.08 ± 8.01 ^**a,b,c**^
LDL (Friedwald)	120.76 ± 34.32	120.06 ± 25.17	120.41 ± 29.85	116.47 ± 20.58	126.25 ± 39.28	133.16 ± 28.24	137.14 ± 34.45	128.21 ± 31.54

Results are presented as means ± S.D. Significant results are presented as bold. The *p*-value indicates significant difference between (a) GG vs. TT individuals, (b) GT vs. TT individuals, and (c) GG + GT vs. TT individuals according to the Welch’s two sample *t*-test (*p*  <  0.05). Abbreviations: WHR, waist-to-hip ratio; BMI, body mass index; HbA1c, glycated hemoglobin; HOMA-IR, homeostasis model of insulin resistance; HDL, high density lipoprotein; LDL, low density lipoprotein.

**Table 5:** Genotypic and allelic frequencies *APOA2* rs3813627 polymorphism.

Condition/Models	Codominant			Dominant		Recessive		Log-Additive	
	OR (95% CI) _het_	OR (95% CI) _hom_	*p*	OR (95% CI)	*p*	OR (95% CI)	*p*	OR (95% CI)	*p*
HDL									
Model 1	0.64 (0.24–1.74)	3.09 (0.85–11.21)	0.065	1.02 (0.43–2.45)	0.956	3.76 (1.11–12.71)	0.030 *	1.42 (0.77–2.62)	0.256
Model 2	0.62 (0.22–1.69)	3.03 (0.81–11.29)	0.066	0.99 (0.41–2.40)	0.979	3.76 (1.09–13.02)	0.033 *	1.40 (0.75–2.60)	0.288
APOA1									
Model 1	0.45 (0.12–1.75)	2.77 (0.71–10.88)	0.055	0.97 (0.33–2.86)	0.952	3.89 (1.10–13.81)	0.035 *	1.49 (0.74–3.03)	0.265
Model 2	0.46 (0.12–1.76)	2.82 (0.71–11.12)	0.054	0.98 (0.33–2.89)	0.964	3.94 (1.11–14.08)	0.034 *	1.50 (0.74–3.06)	0.257

Multiple logistic regression analysis of the *APOA2* rs3813627 polymorphism was carried out in overweight/obese individuals (BMI ≥ 25 kg/m^2^), taking the *APOA2* rs3813627 variant as independent variables an HDL (*n* = 91) and APOA1 levels (*n* = 65) as dependent variables. The *APOA2* rs3813627 GG genotype was considered as the reference category. Model 1 was carried out without adjusting and Model 2 was performed after adjusting by sex (* *p*  <  0.05). The multiple logistic regression analysis was analyzed to determine the risk associated with low HDL and APOA1 levels. Abbreviations: BMI, body mass index; HDL, high density lipoprotein; APOA1, apolipoprotein type A1; OR, odds ratio; CI, confidence interval.

**Table 6:** Transcription factors and motifs overlapping the rs3813627 *APOA2* SNP.

SNP	Alleles	Location	RegulomeDB	HaploReg	rSNPBase	PROMO	SNP FuncPred
**rs3813627**	G/T	161225358	TBL1XR1, AR, HSF1	GR, HSF, PAX-5	HEY1, MYBL2, POL2, CTCF, NFκB, CTBP2, ZNF263, HCFC1, SMC3, TCF7L2, ARID3A, MAF, MAZ, EGFP	c-MYB, FOXP3	AR, BRCA, C/EBPγ, DR4, FAC1, GATA4, HSF1, MYB, PAX, HNF4, PPARα, POU3F2, PPARγ, TBP

Data predicted by the SNP functional consequence, by binding with transcription factors. Abbreviation: Transducin beta-like 1 X-linked receptor 1 (TBL1XR1), androgen receptor (AR), heat shock transcription factor 1 (HSF1), glucocorticoid receptor (GR), paired box gene (PAX-5), hairy/enhancer-of-split related with YRPW motif protein 1 (HEY1), MYB proto-oncogene like 2 (MYBL2), polymerase II (POL2), CCCTC-binding factor (CTCF), nucelar factor κ type B (NFκB), C-terminal-binding protein 2 (CTBP2), Zing finger factor type 263 (ZNF263), host cell factor C1 (HCFC1), structural maintenance of chromosomes protein 3 (SMC3), transcription factor 7-like 2 (TCF7L2), T-rich interactive domain-containing protein 3A (ARID3A), proto-oncogene c-Maf (MAF), Myc-associated zinc finger protein (MAZ,) enhanced green fluorescent protein (EGFP), V-Myb avian myeloblastosis viral oncogene homolog (c-MYB), forkhead box p3 (FOXP3), breast cancer (BRCA), CCAAT enhancer binding protein gamma (C/EBPγ), GATA binding protein 4 (GATA4), hepatocyte nuclear factor 4 (HNF4), death receptor 4 (DR4), fetal Alz-50 reactive clone 1 (FAC1), peroxisome proliferator activator receptor alpha (PPARα), POU3F2, peroxisome proliferator activator receptor gamma (PPARγ), TATA-box binding protein (TBP).

**Table 7:** General information about SNPs in *APOA1* and *APOA2* on lipid traits.

SNP	Gene	MAF * (Risk Allele)	Lipid Traits
rs3813627	*APOA2*	0.31 (T)	Susceptibility to low HDL levels [19].
rs5085	*APOA2*	0.18 (G)	Marginally associated with total cholesterol levels and waist-to-hip ratio [36].
rs5082	*APOA2*	0.24 (G)	Associated with BMI and food consumption, [17,37], with lower total cholesterol, triglyceride, Cholesterol/HDL-c ratio and non-HDL cholesterol [38] and regulates postprandial response to a saturated fat overload [23].
rs6413453	*APOA2*	0.10 (A)	Probably associated with weight [36].
rs670	*APOA1*	0.19 (T)	Associated with increased levels of LDL-c and total cholesterol [39], metabolic syndrome [40] and blood glucose levels [41].
rs1799837	*APOA1*	0.01 (T)	Associated with high triglycerides and insulin levels after a high-carbohydrate diet [42].

* MAF in all populations. Abbreviations: high-density lipoprotein (HDL); low-density lipoprotein (LDL); minor allele frequency (MAF); apolipoprotein type 1 (APOA1) and 2 (APOA2).
